# Mapping the Drugged Body: Telling Different Kinds of Drug-using Stories

**DOI:** 10.1177/1357034X20925530

**Published:** 2020-10-23

**Authors:** Fay Dennis

**Affiliations:** Goldsmiths, University of London

**Keywords:** body-mapping, Haraway, injecting drug use, storytelling, the drugged body, visual methods

## Abstract

Drugged bodies are commonly depicted as passive, suffering and abject, which makes it hard for them to be known in other ways. Wanting to get closer to these alternative bodies and their resourcefulness for living, I turned to body-mapping as an inventive method for telling different kinds of drug-using stories. Drawing on a research project with people who inject heroin and crack cocaine in London, UK, I employed body-mapping as a way of studying drugged bodies in their relation to others, human and non-human, in the injecting event. I invited participants to draw their bodies in describing these otherwise hard-to-articulate experiences. Following Donna Haraway, I conceptualise body-mapping as a more-than-human mode of storytelling where different kinds of bodies can be known. Here, I look at three such bodies – sensing-bodies, temporal-bodies and environment-bodies – and argue that it is through being able to respond to such bodies that more hospitable ways of living with drugs can become possible.

The drugged body…[is] a dreary parade of sucked-dry, catatonicized, vitrified, sewn-up bodies […]. Emptied bodies instead of full ones […]. What happened? Were you cautious enough? […] Many have been defeated in this battle.

Deleuze and Guattari (1987: 150)

As in Gilles Deleuze and Félix Guattari’s uncharacteristically determinist depiction, drugged bodies, that is, bodies that have become-with drugs, are seen to lose their connectivity with the world in becoming focused and reliant on this one connection. Ironically, employing what could be considered a Deleuzo-Guattarian methodology of mapping (Coleman and Ringrose, 2013), this article instead argues that it is through such a method that drugged bodies can make themselves known in new ways based on a more-than-human relationality and mode of living beyond a range of dichotomies that usually confine and curtail them. Method in this sense gets involved in the world it once merely studied (Law, 2004; Lury and Wakeford, 2012; Marres et al., 2018). Aware of the drug-using body as a heavily mediated body (Malins, 2004; Malins et al., 2006; Raikhel and Garriott, 2013; Weinberg, 2002, 2013) and the perils of this kind of representational work (Malins, 2011; Vitellone, 2011), where people who use drugs are surrounded with images of what it is to be a drug user, addict or worse still ‘junkie’, we need different kinds of methods that can tell different kinds of stories. ‘We need other kinds of stories!’, Haraway implores us in the recent film, *Donna Haraway: Storytelling for Earthly Survival* (Terranova, 2016). Trying to get away from these dominant representations, I use body-mapping as a way of focusing in on the embodied here-and-now of what is happening in the drug-using event to move below and beyond abstraction and totalising systems of thought.

In social studies of drug use, the body has often come second to the drug-using mind (Weinberg, 2002, 2013). Disrupting assertions of drug use as an ‘ineffable experience of the body’ (MacLean, 2008), this article instead asks what a methodology centred on the body might do for knowing drug use and the drugged body otherwise. Following a discussion of both the drugged body as an ‘absent presence’ and the traditions of body-mapping, I home in on the method used. More than representation, I explore body-mapping’s potential for wordly repatterning (Haraway, 2016). This gets fleshed out through a discussion of three bodies – sensing-bodies, temporal-bodies and environment-bodies – that emerge through a collaborative act of mapping. It is through such bodies that I pay attention to new kinds of drug-using stories and the possible ways we might respond in making more liveable futures with drugs.

## Background

### The Drugged Body

The body in social studies of drug use (including alcohol), like sociology more broadly (Shilling, 2003, 2012; see also Blackman, 2008), has been noted as an absent presence (e.g. Harris, 2015; Weinberg, 2013). In sociology, Shilling accounts for an enduring Cartesian dichotomy and dual dynamic where the body is ‘absent in the sense that sociology has rarely focused in a sustained manner on the embodied human as an object of importance in its own right’, while being ‘at the very heart of the sociological imagination’ (Shilling, 2012: 17). In drug studies, this absenting has taken at least two trajectories. First, much like Shilling says, the body is treated as a symptom rather than a mode of analysis, and, second, quite purposely, it is neglected as a secondary effect of the rational drug-using mind. Body-mapping is employed here not only to make previously absented bodies present but to get to know different kinds of embodied and more-than-human ways of living with drugs so they may be able to exist more amicably with the processes and forces that try to curtail them.

The first way bodies have been absented in social drug studies follows Shilling’s observation that although the body has always been present, it has tended to be treated ‘as *symptoms*, rather than analyses’ (Shilling, 2012: 14 original emphasis). That is, drug-using bodies have been explored discursively for how they are inscribed and disciplined. The body becomes a site for neoliberal governmentality forces that act to control and censure drug-using bodies through public health strategies and technologies such as the needle and syringe programme (McLean, 2011) or opiate-substitution therapy (Bourgois, 2000; Fraser, 2006; Harris and Rhodes, 2013; Meyers, 2013; Valentine, 2007; Vrecko, 2016). Addiction treatment facilities are similarly seen to operate through a system of re-inscription, producing ‘normal’ drug-free citizens (Carr, 2011; Nettleton et al., 2013; Sedgwick, 1993; Weinberg, 2005). And women’s bodies are particularly targeted (Campbell and Ettorre, 2011; Ettorre, 2007; Knight, 2016). Furthermore, the battered and bruised body of the drug user comes to embody and represent a failing political economy (Bourgois, 2003; Garcia, 2010; Singer, 2006), with these subjugated bodies getting dramatically depicted in visual ethnographies (Bourgois and Schonberg, 2009; Parkin, 2014; Parkin and Coomer, 2009). In this vein, representations of the drug-using body have often focused on the suffering and abject body, producing a body that is done to rather than doing (for critiques, see Harris, 2009; Vitellone, 2011; Weinberg, 2013).

The second way bodies have been absented is rather more purposely in the making present of the rational drug-using mind. Challenging scientific and media representations, for example, of the ‘out-of-control’ drug user, sociologists for a long time have privileged drug use as a meaningful and socially useful practice (Becker, 1953; Young, 1971). More recently, Fiona Measham (2004) coined the term ‘controlled loss of control’ to capture the rationality behind seemingly out-of-control drug-using practices in the night-time economy. The ‘“controlled loss of control” is a calculated hedonistic act which aims to achieve a desired, structured and controllable altered state of intoxication, by pharmacological or behavioural intervention’ (Measham, 2004: 343). Terms like this have since proliferated to make sense of many drug-using practices including those that are otherwise understood as involuntary and compulsive (McGovern and McGovern, 2011). While these analyses are indeed noble in challenging the image of the irrational drug user, they fail to engage adequately with the embodied, affective and other-than-rational forces at play, which can drive desire and connectivity in the drug-using event (e.g. Bøhling, 2014; Dilkes-Frayne, 2014; Moore, 2008; Poulsen, 2015; Race, 2014; see also Dennis, 2019, for a more detailed account of these trends).

In this project, I turn to body-mapping not only as a way of studying the neglected drugged body but as a way of telling different kinds of drug-using stories that eschew dichotomies of mind and body, control and out-of-control, human and non-human. But this is by no means a unique move and reflects wider shifts within the discipline of critical drug studies towards the material, processual and affective nature of drug use, which has drawn extensively from new materialism, posthumanism and Science and Technology Studies (e.g. Duff, 2013, 2014; Fraser et al., 2014; Race, 2018; Seear and Moore, 2014; Vitellone, 2017).

Following these trends, drugged bodies are a matter of sociomaterial ‘intra-action’ (Fraser and Valentine, 2008; Malins, 2004) – pre-individually relational – made up of the very processes that might have once been seen as outside or above them (Weinberg, 2013). In this sense, the ‘drug’ in the ‘drugged body’ does not necessarily refer to or rely on the physical drug per se but the drugging effect of these interrelating processes, which indeed may not need the drug at all (Fraser and Moore, 2011; Keane, 2002; Malins, 2017). These trends of course build on a long line of body studies that have sought to disrupt the body as given and explore what the body can do in its relationality with others, human and non-human, and with this, its potential for disentangling and reorganising itself and worlds (e.g. Bourdieu, 1984; Butler, 1993; Dewey, 1934; Mauss, 1934). It is this active and relational body, or what Bruno Latour (2004), in this journal, calls the ‘interesting body’, that has captured my and many drug scholars’ imagination.

However, so far, beyond observation, there are few methods that attempt to capture and intervene in this lively embodied construction. Here, I develop the method of body-mapping to make a previously abstract concept of the sociomaterially drugged body-we-do more empirically knowable. In enacting new kinds of stories, the method stays true to its origins as a mode of storytelling, but where body-mapping-as-storytelling has been conceived as an epistemological matter of representing partial truths, I also think about it as a mode of intervention, for engendering more hospitable ways of living with drugs in these hostile times.

### Body-mapping

Body-mapping has taken many forms, but broadly speaking, researchers in the social sciences have used it to refer to a method that seeks to explore embodied experiences using storytelling tools that centre on the human body. Body-mapping has traditionally involved life-size paintings of the body and originated as an art-therapy and research device for people living with HIV/AIDS (Art2Be, n.d.; Brett-MacLean, 2009; Devine, 2008; MacGregor, 2009; MacGregor and Mills, 2009; Nöstlinger et al., 2015; Orchard et al., 2014; Solomon, 2002). However, it has since been taken up in a range of settings and to describe an array of visualising methods (e.g. Cornwall, 1992; Crawford, 2010; Lys et al., 2018; Naidoo et al., 2014; Shanneik, 2018; Tarr and Thomas, 2011; Tarr et al., 2014). Its wide uptake speaks to the method’s diverse historical roots in many cultures as an anatomical diagram and the tradition of visualising the human body as a way of knowing the world (Kidel and Rowe-Leete, 1989; for a wider discussion on how visualisation practices such as maps and diagrams have informed knowing, see Grasseni, 2007).

In the first methodological guidebook of its kind, ‘*Body-Map Storytelling as Research*’, Gastaldo and colleagues argue that ‘“body mapping” is the process of creating body-maps using drawing, painting or other art-based techniques to visually represent aspects of people’s lives, their bodies and the world they live in’ (2012: 5). They continue: ‘body mapping is a way of telling stories, much like totems that contain symbols with different meanings’ (2012: 5). Taken up in subsequent studies, this manual has formed an instructive way forward for the methodology as a partial practice of representation (Gastaldo et al., 2018; Mason, 2018). In my project, however, and drawing on wider methodological debates, especially within human geography, towards the non-representational (McCormack, 2014; Thrift, 2007), I sought to engage the method in the non- and more-than-representational aspects of life and living.^1^ That is, rather than being interested in how body-mapping can represent life themes and meaning-making, I explore what it *does* in and for the research encounter.

## More than Representation: Storytelling as Repatterning

In this article, I analyse body-mapping as a more than representational mode of storytelling in worldly repatterning (Haraway, 2016). Rather than ascribing to any set meaning of ‘more-than-representation’ (e.g. Lorimer, 2005), I draw from a long line of body studies (e.g. Merleau-Ponty, 1954) that work with the body as doing. That is, the body is always to some extent determining its own agency and meaning beyond that which is consciously known, and actively engaging with those practices, affects and discourses that were once thought to produce it (Blackman and Venn, 2010). In this section, I explore this mode of storytelling as a potential pathway through otherwise pathologising narrativising technologies that try to limit the drug-using experience.

Drug consumption and addiction are highly ‘storied’ practices and ways of being (e.g. Carr, 2011; Fraser et al., 2018a; Keane et al., 2011; Pienaar and Dilkes-Frayne, 2017; Pienaar et al., 2015; Raikhel and Garriott, 2013; Reinarman, 2005; Weinberg, 2000). Powerful discourses, coming from psychiatry (Fraser et al., 2014; Keane et al., 2011), the media (Fraser et al., 2018a; Malins, 2011), policy (Fraser, 2015b; Lancaster et al., 2017; Seear, 2019) and popular culture (Boothroyd, 2006; Manning, 2007), through biographical writing (Fraser, 2015a; Keane, 2001), social media (Dwyer and Fraser, 2016) and television programmes and films (Vitellone, 2004), work to construct drug use and addiction as an individual problem of the drug-using subject, or, increasingly, the brain (Fraser et al., 2018b). Frequently conflating the two, drugged subjects are seen to be unable to exercise self-control and volition, acting out of a physiological need, which only too readily neglects drugs’ pleasures and sociality. Indeed, such is the power of these discourses that any recourse to perceived positive experiences can be reduced to denial and ‘addict speak’ (Moore, 2008; O’Malley and Valverde, 2004; Pienaar et al., 2015)

To go below and beyond these representations, I use mapping as a way to pay attention to the here-and-now of the drug consumption event, rather than what it might mean to the consumer outside of the situation, or indeed, to the audience in viewing the images. Perhaps, because of the method’s history in art-therapy, and the use of ‘projective drawing’ in psychology where colour, size and other aspects of drawings have been connected to certain psychological traits and personalities (Bagnoli, 2009), I was wary of the kinds of interpretations the body-maps could afford. Drawing on what we have now known for some time, that methods get involved in the world (Back and Puwar, 2012; Coleman and Ringrose, 2013; Latour and Weibel, 2005; Law, 2004; Law and Urry, 2004; Lury and Wakeford, 2012), I became more interested in what the body-maps could *do* and the different kinds of stories they enabled to be told.

In Donna Haraway’s (2016) ‘Playing string figures with companion species’, she reinvigorates storytelling as a more-than-human practice of return and relay. Using string figuring as her metaphor, she describes the giving and receiving of patterns to not only tell stories but construct more liveable futures.

Playing games of string figures is about giving and receiving patterns, dropping threads and failing but sometimes finding something that works, something consequential and maybe even beautiful, that wasn’t there before, of relaying connections that matter, of telling stories in hand upon hand, digit upon digit, attachment site upon attachment site, to craft conditions for finite flourishing on terra, on earth. (Haraway, 2016: 10)

In this article, I conceptualise body-mapping as a kind of string figuring of bodies, objects, things and knowledges. By giving equal weight to these entities and forces, a rescaling occurs where non-human objects become just as important as human subjects. In one telling moment, a participant exclaimed that he had forgotten ‘the car’ (discussed below). Rather than the drug dealer, he had forgotten their car – a constant go-between and reminder of drugs. Reorganising what Haraway calls ‘contested subjects and objects of “modern progress” and “backward tradition”’ (2016: 15), this kind of mapping as storytelling is able to reinvigorate how people live with drugs, where drugs and other non-human others have been neglected in favour of the drug-using subject (Duff, 2012). Much like the capabilities of the pigeons in Haraway’s (2016) multispecies storytelling (explored further below), drugs ‘surprise and impress human beings, who often forget how they themselves are rendered capable by and with both things and living beings’ (2016: 16). Taking all kinds of materiality seriously, let me now move to how this more-than-representational storytelling played out in practice.

Employed in the interview process, body-mapping provided a *closeness* and *movement* between bodies, non-human objects and knowledges. It afforded a less clinical atmosphere, where embodied performativity is brought to the forefront (Ezzy, 2010; Harris, 2015). The shift from exclusively talking to drawing provided the space and time to stand-up and move about (e.g. check phones, get a drink, open/close windows, rearrange the tables and chairs) and then move in closer for the drawing. The movement offered a chance at ‘performing embodiment’, in Gaudelius and Garoian’s (2007) terms, to ‘rupture [the] body’s enclosure in order to cross the threshold that separates [the] body from the world [-] the “skin” of Cartesianism, which separates our understanding of the body, language and technology’ (2007: 4). The quiet periods (during drawing) allowed more time to create and take in the affective atmosphere, and in particular, participants’ affective responses to their drawing, including mumblings, singing, whistling and sighing. The drawing itself then acted as a prompt for remembering and diffracting meanings throughout the interview.

In a string figuring of bodies, discourses, drawing materials, furniture and so on, new bodily formations were made knowable. That is, in a venture of joint problem-making, these collective bodies could push against some of the dominant ways bodies are usually ‘absented’ or made (un)knowable in recourse to pathology and thus make different kinds of bodies and the stories they tell matter. To quote Haraway: ‘All together the players evoke, trigger, and call forth what – and who – exists’ (2016: 16).

## Mapping Bodies: Telling Different Stories that Matter

When social scientists find and map the capture, fixing and unfixing of the body and its machinic connections they offer hope and possibility for something different in the social. (Coleman and Ringrose, 2013: 142)

Drawing on repatterning as an ontologically more intimate approach to bodies than representation, I now attend more closely to the maps themselves and the kinds of bodies and stories they render possible. Through these often elaborate and intricate drawings, participants depict and amplify various bodies and capacities for embodiment. Elsewhere, I have looked at these in the ways bodies become ‘normal’ (Dennis, 2016a), ‘triggered’ (Dennis, 2016b) and some*body* else (Dennis, 2017). Mapping-as-storytelling redraws normative bodily boundaries, and as such, what the drugged body *is* and can be. Most notably, the drugged body gets repatterned beyond the afflicted and passive ‘addict body’ that is submissive to the workings of the habituated mind (Sedgwick, 1993) or, more recently, the chemically altered brain (Fraser et al., 2018b; Vrecko, 2010). By disrupting hierarchical and dualistic thinking, mapping bodies makes new kinds of bodies knowable. Here, I attend to these previously absented bodies as ‘sensing-bodies’, ‘temporal-bodies’ and ‘environment-bodies’.

### Sensing-bodies

**Figure 1.**
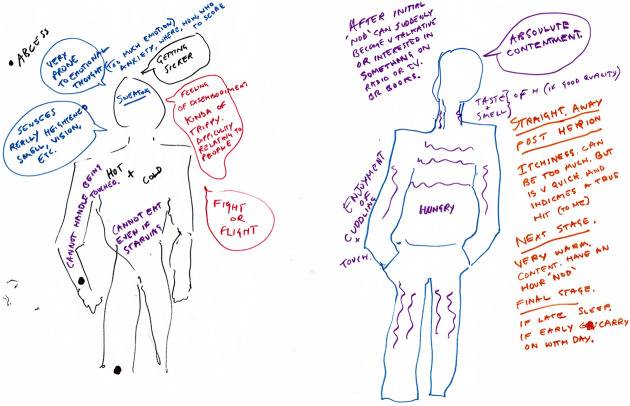
Simon’s body-map highlights some of the sensory and affective qualities of injecting heroin (left: before injecting and right: after injecting).

**Figure 2.**
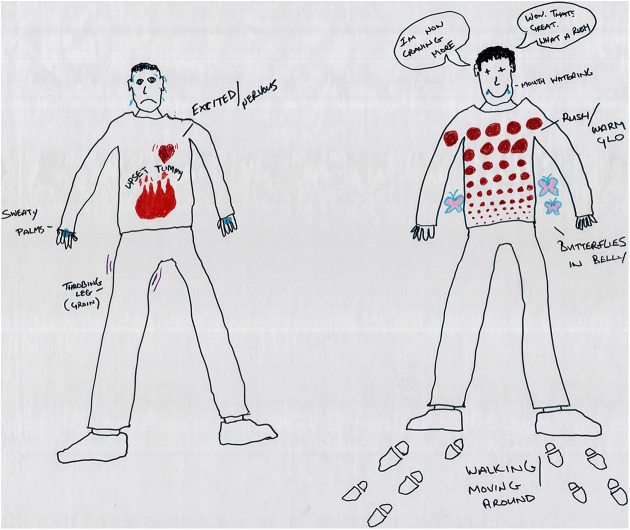
Mike’s body-map shows some of the feelings associated with injecting heroin and crack cocaine (‘speedballing’) (left: before injecting and right: after injecting).

Being asked to think about embodied experiences is not an everyday practice, and indeed, one participant said back to me, questioning my question, ‘how does it feel?’, as if nobody had asked him that before. In this sense, body-mapping worked to ‘orient’ (Blackman and Venn, 2010) participants towards their bodies, towards the sensual and affective, as a ‘visceral prompt’ (Hickey-Moody, 2013). This responds to old and, worryingly, ongoing calls from within critical drug studies to pay more attention to the body (e.g. Fraser et al., 2018b; Harris, 2009, 2015; Malins, 2004, 2017; Weinberg, 2002, 2013). Body-mapping prompted participants’ bodily experiences of injecting drugs with an awareness to smell, touch, taste, vision and the affective. For example, heroin and crack cocaine are two substances not usually thought about in terms of their taste but were interestingly valued by participants in these terms. Taste could indicate the authenticity of the drug and pre-empt ‘the rush’, while also making it enjoyable in its own right – what one participant referred to as ‘tasty’ rather than ‘taste’. Simon and Mike’s body-maps (Figures 1 and 2), in particular, discuss some of these sensory and affective aspects of injecting heroin and crack cocaine.

We see in Figure 1 how Simon illustrates the sensory aspects of injecting heroin by comparing before he injects, where, among other feelings, he feels ‘hot and cold’, ‘cannot handle being touched’ and has a ‘heightened’ sense of smell and vision, to after injecting heroin, where he gets an immediate ‘itchiness’, followed by a ‘warmth’ and an ‘enjoyment’ of touch and ‘cuddling’ and an increased capacity for ‘interest in something on the radio or TV or books’. That is, in relation to the drug as well as those entities that make its effects possible – the vitamin C (which induces the itchiness), human others (for cuddling) and the radio, TV and books – he expresses a fluxing capacity to engage in touched, smelt, heard, visualised and imagined worlds. Such drugged networks play a crucial role in holding the sensory body together, which tells a very different story to the dominant addiction one that focuses on the destructive nature of drugs where bodies become compelled and controlled.

Simon says, ‘I haven’t really talked about this before, but it might interest you: “disembodiment” [he writes]’. He goes on, prompted by the pen in his hand and resurging bodily memories that are never too far away, to explain how drugs help to contain his senses, of which before he uses, he feels unbounded and outside of his body – uncontained:

Everything is all ‘oh oh’ [makes sounds and gestures to show how things get too close] like that, everything is like right, like if you’re trying to walk around the streets and it’s just like you can’t handle busy high streets and you know busy tubes and….

There is a sensory overload in which the outside world moves in and becomes too close for comfort. For Simon, heroin helps keep his distance and enforce a bodily barrier to feel part of the world rather than enveloped and subsumed by it. Like Howes (2018) analysis of ‘skinscapes’, this is a more-than-human sensuous epistemology for engaging with rather than closing off from the world.

Another participant tells a similar story. Mapping out her experience of taking diamorphine, she says: ‘I can get on with being normal, more better […] It just helps me cope with everything’. And in an act of relay, she is pleased with an expression I use: ‘I think you’ve just helped me there by saying it takes the edge off things, I’ve been trying to put that into words for a long time, I didn’t know how to say it’. Allowing to be moved by the mapping process, new ways of knowing drugs and drugged bodies are made possible.

Getting a deeper sense of how the drawing materials, bodies and knowledges work together in the mapping process, Mike (Figure 2) is oriented to the sensory body and its capacity to affect and be affected. Affording this matter-of-fact talk that circumvents hypothesising and judgement, he explains – mapping as he thinks and thinking as he maps – how he gets ‘excited/nervous’, with ‘sweaty palms’, but also sometimes gets a ‘throbbing leg’ (from an injecting site) and ‘upset stomach’ (shown through what he calls a ‘fire in my belly’). When he is about to inject, he gets ‘butterflies’, and then when he has injected himself, he experiences a rush through the body which starts at the waist and moves up to the head (depicted by his meticulous drawing of dots that slowly increase in size). At this point, the throbbing pain is alleviated, his eyes go into crosses to show he is intoxicated and his mouth relaxes into a broad smile, drooling slightly at the edges.

Sometimes, when you have the crack, your mouth starts to water as well, you start to salivate. It’s weird. You can literally start dribbling. It can mess up your speech and everything if it’s really that strong…Right, so sometimes I can become quite restless, quite fidgety, and walking around and that [thinking about how to depict it]…I’ll just draw footsteps.

Orienting to bodies and their capacities to affect and be affected, we get a sense here of the ways bodies have learnt to become-with drugs, becoming anxious in their absence, but once injected, re-energised; they incorporate and ‘excorporate’ (Mol and Law, 2004), extending out, leaking fluids, becoming fidgety and walking around. Sensing-bodies disrupt the boundaries between non-humans and humans, inside and outside (Urry, 2000). Senses work at the intersections of human and non-human processes and register affect modulation as bodies become able to feel and do new things in relation to others. For ethnographer of affect, Kathleen Stewart (2007), a subtle and nuanced approach of ‘noticing’ is thought necessary in exploring the everyday charges, intensities and eruptions in bodily rhythms and feelings that go beyond sensory responses. Mapping more-than-human bodies can similarly be thought of as a way to draw attention to the invisible and immaterial forces, or the draws and pulls between bodies and that which move bodies beyond addiction. For example, through the drawings, participants noted how drugs were able to produce affects beyond their pharmacological interaction with ‘the body’. One participant described an induced calmness in only holding heroin – ‘Once you’ve got it in your hand you start to feel better, as soon as you’ve got it’. There is a collaborative knowing between the drug and the body that relief is coming. This allows a way in ‘beside’, as Eve Sedgwick (1993) would say, the dominant biomedical model of addiction, which requires the substance to physically enter the body to ‘feel better’.

Far from dumb (as in narratives of the addict body), sensing and affective bodies tell stories of joint learning, holding together, maintenance and care. Therefore, much like Stewart’s rejection of ‘totalized systems’ in everyday America, mapping sensing-bodies in this way brings drug-using bodies ‘into view as a scene of immanent force, rather than leaving them looking like dead effects imposed on an innocent world’ (2007: 1).

### Temporal-bodies

Bodies are not something we have, as ‘fixed’, but are actively held together or capacitated in their movement or bodily routines and rhythms with others. This is essential for understanding the temporality of drug use and drug-using bodies, which often gets understood by the participants as habit. As Simon (introduced earlier) puts it:

I see it as a habit, there is a lot about getting up, getting to the time that they [drug dealers] switch on, going out and getting the stuff and coming back and having a…you know, having a use, that’s, a lot of that is habit, just cos *it’s what I do* […] It’s a funny, it’s a very strange relationship, you do almost feel, there is a quality where it’s almost like a friend, you know, that it’s quite comforting sometimes, the feeling is very comforting. (emphasis added)

Simon explains that a lot of his drug use is out of habit. *It is what he does.* This stresses the drugged body as something we *do* as it goes along (Massumi, 2004). There is a momentum to his words which suggests the rhythms of this work: ‘there is a lot about getting up, getting to the time that they switch on, going out and getting the stuff and coming back and having a…you know, having a use’. Simon brings to our attention how such habits have become part of him – a new way of being-in-the-world – which, as he explains, makes them hard to give up. Habit, as a central concept in body studies, allows us to appreciate this important work, what Simon describes as friend-like. Habit disrupts ‘any clear and distinct boundary between nature and culture, self and other, the psychological and social, and even mind and matter’ (Blackman, 2013: 186). Habit becomes embodied and, in modulating affect, encompasses a dual role of both regulating and creating bodies (e.g. Blackman, 2013; Grosz, 2013; White, 2013). Therefore, it seems that in their very doing, as temporal matters that need to be held together, drugged bodies become repeatable, carrying capacities, but in this have the ability to change.

**Figure 3.**
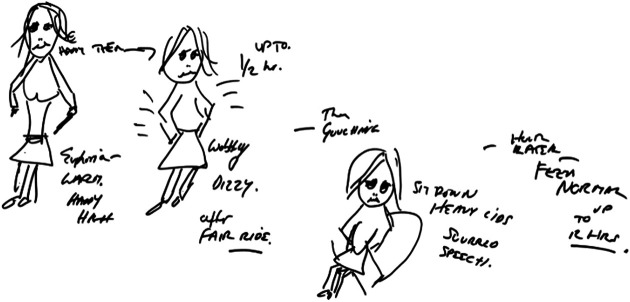
Gwen’s body-map shows temporal-bodies in their making with heroin and another non-human entity, the chair.

**Figure 4.**
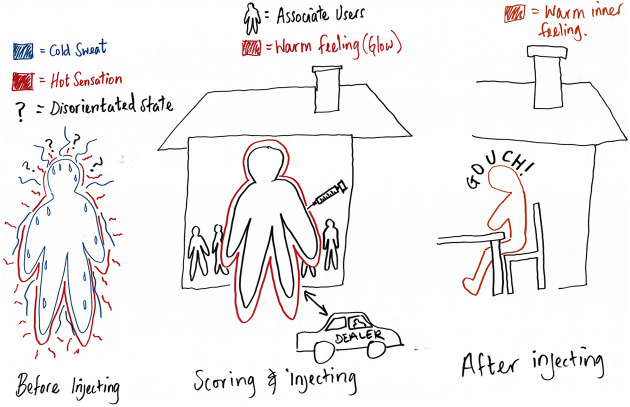
Reggie’s body-map shows temporal-bodies connecting with the car, ‘crack house’ and chair.

Gwen’s body-map (Figure 3) depicts some of the bodily changes wound up with time and repeatability. Time, like the body, gets made and remade in these connections with drugs, in her case, injecting heroin. She describes ‘the stages of the drug’, which orders the temporal body: taking her from being ‘happy, euphoria, warm, happy heart’ (10–15 min), to then feeling (up to half an hour) ‘wobbly, dizzy’, which she compares to coming off a boat or fairground ride, to then, ‘gouching’ [feeling the sedating effect of heroin] with ‘heavy eye lids’, but notably this is only when she sits down, to ‘slurred speech’ (1 h), to then, ‘feeling normal’ (up to 12 h). Interestingly, time does not objectively measure these feelings but rather is intimately involved in making these bodies, in connection to other bodies, for example, the chair (drawn), which affords gouching. It is only when sitting down, she says, that long periods of gouching are possible. This is a notion also expressed by Reggie (Figure 4) in relation to the ‘crack house’ and its inhabitants that he says can substantially reduce the gouching period: ‘If I’m with people in a crack house, I don’t really know them and that, then I can’t relax in there’. He moves from the second to the third scene, going back to his own house to enjoy the gouch. Another participant, Vicki, similarly stresses how gouching is relationally constituted and enacted.

The gouching part is only if you are sitting there and you’re relaxed. If you’re up doing something, it’s not like it will make you sit there and gouch, it doesn’t do it like that, it depends like what you’re doing.

Contrary to addiction narratives of compulsion and the overpowering pharmacology of drugs acting *on* the body, mapped drugged bodies tell a different story. Far from drugs simply causing intoxicating effects, the effects are contingent. Body-mapping makes us pay attention to the non-human objects and things that so often get ignored – the chair, crack house. In another example, as aforementioned, Reggie, in realising he’d left something out, exclaims: ‘aha, I’ve missed the car’. Rather than the individual drug dealer, who comes and goes, he had missed the car – an ever-present object in his local area that affectively triggers a desire to consume drugs. Mapping bodies, objects and knowledges together produces a flatter scale in which objects do not simply come to stand in for more meaningful subjects or represent bigger scenarios but can be taken seriously in their own right, intimately involved in the doing of the drugged body.

These drugged routines and rhythms change capacities to affect and be affected and thus precede individuation. However, there is a paradox, just as movement seems to get locked into bodies, new movement is created. As rhythms get made and become imprinted they become unmade – there is always difference in repetition. Or, as Deleuze famously says, ‘difference inhabits repetition’ (1994: 76). This is an inherently more optimist approach than addiction, which gets drawn attention to in Nadiya’s account.

I think if you stop, when you wake up in the morning, it’ll be the first thought, but about two or three weeks down the line, you wake up some mornings and you won’t think about it, you know, you’ll think about something else. A lot of time needs to pass for you to stop, and I think that’s the worst part because in the meantime, you’ve got really *nothing to do*, and people aren’t really going to take up menial jobs or I don’t know. It’s a vicious cycle. emphasis added

There is the potential for something different. Nevertheless, this is no easy task and Nadiya cautions: ‘a lot of time needs to pass for you to stop’. However, crucially, for this to happen, she needs something else to *do* to pass the time. Time is not an abstract phenomenon (an objective measure) but reliant on what we do and the ‘things’ used to navigate its passing (topologically). By seeing bodies in these temporal terms, they are always changing, but this relies on more than the ‘doing’ of the person who uses drugs, such as the availability of interesting jobs (and one can imagine a long list of activities that foster meaning and value), to bring about the time needed to change.

### Body-environments

**Figure 5.**
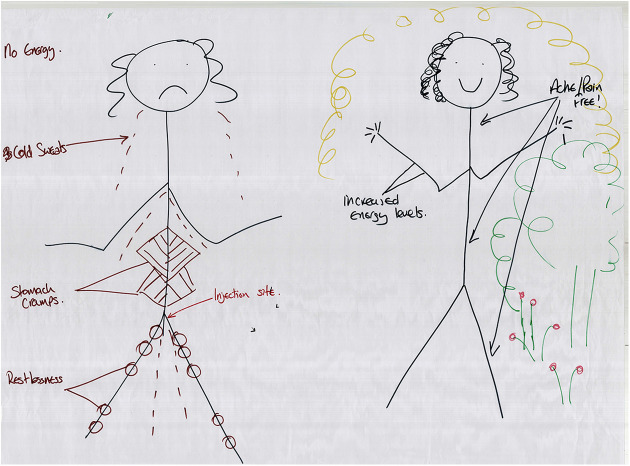
Vicki’s body-map attends to her capacities to notice the ‘natural’ environment when ‘feeling well’.

As already seen through the body-maps earlier, bodies are made and moved in relation to others. As such, the body-maps highlight how ‘bodies’ cannot be separated from their environment. Environments become an integral part of the drugged body (Deleuze and Guattari, 1987, see also Rosengarten and Michael, 2009), and their capacity to affect and be affected in the drug-using event. Several participants draw attention to the role of their ‘natural’ surroundings in their positive experiences of consuming drugs. In drawing trees, Vicki (Figure 5) says ‘I try to notice them when I’m not feeling well [withdrawing from heroin] but I always notice mother-nature more [when I am well]. When I’m feeling better then, y’know, you notice everything’. In mapping her feelings in this way, she is pushed to think *with* these things otherwise seen as mere background. Another participant also highlighted the importance of the sun in whether he was able to ‘cope’ with withdrawing from heroin or not, highlighting the extent to which these somatic ‘inner’ feelings are heavily contingent on the ‘outside’. It is through this back-and-forth between bodies and environments that we get to know drugged bodies, in this example, as attentive and at-peace (if only temporarily).

**Figure 6.**
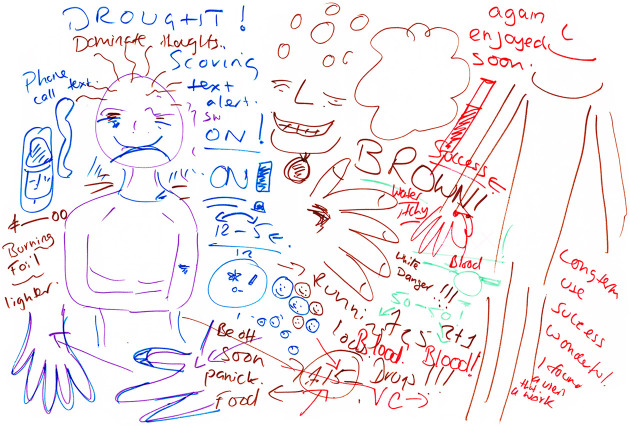
Lucy’s body-map depicts the entanglement of body-environments.

This fluxing ability to feel-in-the-world is similar to Simon’s account of feeling too close to the world, which is a theme that gets repeated. Like Vicki’s golden arch curving around the picture of herself on the right hand side of Figure 5, another participant, Lucy (see Figure 6), draws a bubble to express how a coming together of precarious things in the injecting event can make her feel safe and secure. While all the maps disrupt a concept of the autonomous subject, perhaps none do so as dramatically as Lucy’s (Figure 6).^2^

Through the mapping, we are confronted with the fact that Lucy is not acting alone. To give a sense of how this thinking-as-making and mapping-as-thinking worked, Lucy says: ‘I should really draw a hand more than anything, because when I think of [injecting]…so that would be the area. Round the thumb area. So that’s the area [shows me her thumb]’. In such moments of intimacy, her body, my body and the drawing materials relay to produce these representations. She is then reminded: ‘what I always do is washing up to bring them up’. Doing the washing up (of dishes) is very much part of the event to make a vein viable for injecting. And then jumping back in time and across space, to before the injecting event, as her body remembers and jerks into action:

and then a text [from a dealer] has come along […] And it will go from me being that way [smiling face] to me being sad [reverses the smile on the face], cos then I’ll start thinking about “scoring”.

The mobile phone is a crucial part of this new connection. Indeed, it goes off in the interview with dealers’ ‘adverts’ and panic-inducing messages. This then prompts Lucy to write the word ‘drought’ in bold, which as a memory is never too far away, especially when dealers’ messages say things like ‘running low’. A year ago, Lucy recounts, due to the destruction of the opium poppy fields, ‘the dealers got so much control cos no one could get hold of anything’. Mapping in this way, international drug policies become embodied as a loss of control, shaping response-abilities, where ‘things and living beings can be inside and outside human and non-human bodies, at different scales of time and space’ (Haraway, 2016: 16).

Imagining the threads of a string figure hanging off the body parts, objects and things drawn, body-environments tell stories of connectivity. Far from the body interacting only with the drug, there is a whole host of things involved in thinking and living with drugs. Representation in this sense is working as more than representation. Stories do not only give accounts but refigure previously figured out and fixed bodies. Bodies are no longer submissive, environments passive and drugs all powerful, but they work together in producing the drugged body that is always on the go and subject to change. Weaving together body parts, ‘natural’ surroundings, dealers, mobile phones, adverts and political interferences, mapping tells a complex story of want and desire that disrupts and complicates the usual stories of compulsion or volition.

## Drawing Things Together

The embodied stories told here matter in that they allow other bodies to matter. Re*drawing* boundaries beyond the human/non-human, space/time, outside/inside, new kinds of bodies can be known and made knowable, including what I have called sensing-bodies, temporal-bodies and body-environments. Body-mapping thereby opens up these new ways of doing bodies with drugs. As such, this article has argued that body-maps are better analysed as a mode of more-than-representational storytelling, and thus, neither can they nor do they seek to capture what they attempt to depict. In this move, they call into question their own involvement in what gets made in research. Body-mapping brings human bodies, non-human objects, substances, technologies, political interventions and knowledges into new kinds of relationships. Here, we have seen how these collective bodies, for example, hold together and care for the body-in-flux, potentialising change as they repeat and implicate and depend on that which is usually considered ‘outside’ them in initiating drugged desires and effects. In this concluding section, I discuss the capacity of body-mapping for invention in terms of its experimental and speculative potential for bodies to become-with drugs differently.

To return to Haraway’s (2016) imperative to tell different kinds of stories and, specifically, to her example of taking pigeons as her companions in storying environmental justice otherwise, I want to explore the ways body-mapping brought new relationships into being and, as such, could be considered a site for experimentation and doing politics differently. Like the pigeons escaping their label of ‘rats with wings’ and markers of being ‘unruly’, ‘dirty’ and ‘feral’, drugged bodies, in their mapping, similarly moved from abject to lively, numb to feeling, helpless to hopeful, dumb to thinking. Mapping bodies allows them to tell different stories of themselves in engaging others. Encapsulated in this quote from Haraway:

Why tell stories like my pigeon tales, when there are only more and more openings and no bottom lines? Because there are quite definite response-abilities that are strengthened in such stories. (2016: 115)

There are two key points here, openness and response-ability. Therefore, to detour only momentarily, I want to briefly engage with Waterton and Yusoff’s (2017) discussion of ‘indeterminate bodies’ hosted in this journal. For Waterton and Yusfoff, as well as their contributors, one of the key questions in studying indeterminate bodies ‘in a state of open-ended and affective mutability in relation with [their] world’ (2017: 4) is a methodological question of how to know them in a way that does not close them down, that allows for their openness. Body-mapping in this sense speaks to a wider problem in body studies on how to study bodies in a way that is still open – a problem, I would suggest, particularly for stigmatised and pathologised bodies that are always only too quickly known and shut down. Body-mapping attempts to avoid some of these violences of determination in making indeterminacy visible while generating indeterminacy in an attempt to move us ever closer to the body-in-process and ways of being differently with drugs.

As experimental devices, body-maps open up ways of not only knowing new kinds of bodies but also making them matter through a heightened response-ability. In an extension of the experiment in the natural sciences, Science and Technology Studies scholars have started to use the term to account for the ontological work that their research also does in testing and making things known (Lezaun et al., 2017). But rather than based on truth, social scientific, like scientific matter is made to matter through its sociomaterial apparatus (Barad, 2007). While I have looked at three of the bodies made possible here, these are only some of the many enacted in the interview and that will continue to emerge as the maps entangle with others in discussions, conferences, articles and so on. As an experimental device (Law and Ruppert, 2013), they ‘too are active, alive and lively’. It becomes important, Law and Ruppert argue, ‘to understand that how [devices] establish relations, how they play out, and who and what they mobilise are to a large extent indeterminate and contingent’ (2013: 231–232).

Furthermore, in recognising the role of researchers and methods in co-constituting the world we once merely studied, we can experiment or speculate to create better worlds. Drawing on Haraway, among others, Maria Puig de la Bellacasa (2017), for example, makes a powerful argument for the role of care as we are thrown into new kinds of responsibilities in maintaining more-than-human worlds. That is to say, we need to care for the realities we bring into being through our sociomaterial research practices. As Puig de la Bellacasa puts it, because ‘an ethics of care cannot be about a realm of normative moral obligation but rather about thick, impure, involvement in the world’, it is a ‘hands-on’ ‘ongoing process of re-creation of “as well as possible” relations and therefore one that requires a speculative opening about what a possible involves’ (2017: 6). That is, by heightening our capacities to attune to these collectives, body-mapping affords a responsiveness to these new and potentially more ethical ways of being that resist hierarchical and binary ways of knowing.

Challenging the addict body as dumb and, namely, devoid of or only tangentially engaging with the sensory, time-space and the environment, mapping bodies through an embodied act of prompting and descriptive retelling enables new bodies to be known. For Haraway:

Each time I trace a tangle and add a few threads that at first seemed whimsical but turned out to be essential to the fabric, I get a bit straighter that staying with the trouble of complex worlding is the name of the game of living and dying well together on terra, in Terrapolis. (2016: 116)

Our ability to attune to bodies through body-maps *matters* and sensitises researchers and audiences to these other bodies at play, thus involving them too, by which it exposes ‘possible futures that cannot be managed in advance’ (Savransky et al., 2017: 7). This imagines new ways of doing politics through interventions and policies that can work *with* contingency. For example, policies that take seriously the role of drugs in holding sensory-bodies together may be able to offer substitute medication to avoid the kind of sensory overload and extreme discomfort that Simon depicts. Interventions that take seriously the contingency of time in what the body can do will recognise – at an embodied ontological level – the necessity, as Nadiya describes, of meaningful activities for bodies to learn new habits. And perhaps most obviously and acutely, an appreciation for the ways bodies are entangled within drugged networks could substantiate a more tolerant and empathetic approach to drug use based on difference.

While mapping bodies through drawing in the interview engages those sensing-, temporal- and environment-bodies seen here, new ways of mapping bodies, such as through art workshops, could bring about yet different kinds of bodies and response-ability. As bodies are capacitated and incapacitated in relation to others, including researchers and publics, it is my hope to continue to engage speculatively with these experimental practices in striving to bring into being new ways of living with drugs, to use Puig de la Bellacasa’s phrase, ‘as well as possible’.

## Notes

1. Body-mapping in this study took place within the in-depth interview with 30 people who inject drugs (predominantly heroin and/or crack cocaine). Participants were provided with an A1 piece of paper and a selection of drawing equipment and asked to describe their embodied feelings before, during and after using drugs, and what might be going on around them at those times. For more information on my use of this method and its place in the wider study design, see Dennis (2019).

2. For which reason it is necessarily reproduced here from Dennis (2016b, 2017, 2019).
